# The Physics, Information, and Computation of Perennial Learning: Kolmogorov Complexity, Information Distance, and Port-Hamiltonian Thermodynamics

**DOI:** 10.3390/e28050551

**Published:** 2026-05-13

**Authors:** Chandrajit Bajaj

**Affiliations:** Department of Computer Science and Oden Institute for Computational Engineering & Sciences, The University of Texas at Austin, Austin, TX 78712, USA; bajaj@cs.utexas.edu

**Keywords:** perennial learning, Port-Hamiltonian systems, Kolmogorov complexity, normalized compression distance, curriculum learning, information thermodynamics, Differential Policy Optimization (DPO)

## Abstract

Real-world autonomous agents learn under nonstationarity, safety constraints, and finite energetic budgets. We develop a framework for perennial learning—agents that continuously refine their models while provably controlling the cost of forgetting—by unifying three classical pillars: Kolmogorov complexity, which equates scientific discovery with algorithmic compression; Landauer’s principle, which assigns a minimal thermodynamic cost of $k_{B}T\ln2$ per erased bit to every irreversible model update; and port-Hamiltonian (PH) dynamics, whose (J−R)∇H decomposition separates zero-cost reversible inference from costly irreversible forgetting by construction. The Maxwell demon analogy is formalized: each learning episode is a Szilard cycle in which information acquisition, belief transport, and memory erasure must balance thermodynamically. The information-distance framework, comprising the normalized information distance (NID) and normalized compression distance (NCD), provides a computable geometry for measuring learning progress and guiding curriculum design. We separate theideal uncomputable regularizer based on prefix complexity from the practical compressor/MDL (minimum description length) surrogate that appears in optimization and prove a calibration lemma linking the two under a mild uniform-accuracy assumption. Under explicit regularity, compact-sublevel, and non-energy-extracting assumptions, we prove a passivity speed limit for curriculum-induced contractions of the effective feasible set. Under local asymptotic normality, we reprove that Fisher information is a local posterior codelength proxy rather than an exact theorem about algorithmic entropy. A conditional sequential information-budget proposition shows that the per-stage sample requirement scales as $\tilde{O}\left(\Delta k_{t}/\lambda_{⋆}\right)$, where $\Delta k_{t}$ is the number of materially changed model coordinates (not the total model complexity $k_{t}$); the $k^{3}\to \Delta k$ improvement is conditional on a warm-start assumption and a chosen cold-start baseline. A double-integrator running example with a moving obstacle illustrates the architecture.

## 1. Introduction

### 1.1. Dedication

This article is dedicated to Professor Paul M.B. Vitányi on the occasion of his 80th birthday. It is offered in recognition of his foundational contributions to the theory of computation, Kolmogorov complexity, normalized information distance, compression-based similarity and clustering, model selection and structure functions, reversible computation, and the broad application of algorithmic information-theoretic ideas across computer science, mathematics, cognition, and the sciences [1,2,3,4,5].

This paper follows and attempts to build upon the algorithmic information-theoretic program associated with Li and Vitányi by treating learning as compression, transfer as information distance, and practical adaptation as a computable description-length surrogate for ideal Kolmogorov complexity.

### 1.2. The Need for Perennial Learning

Autonomous agents deployed in the physical world confront an unavoidable reality: the data distribution shifts continuously. Autonomous vehicles must accommodate evolving traffic patterns, weather, and infrastructure modifications [6,7]. In healthcare, wearable devices and adaptive prosthetics must recalibrate to a patient’s changing biomechanics. Industrial predictive-maintenance systems trained on data from new machinery become unreliable as components age. Smart-city infrastructure evolves over decades, with traffic, energy-consumption, and population profiles drifting continuously [8]. Even natural language processing requires models that track cultural and linguistic evolution without forgetting foundational structure.

Perennial learners deployed in consumer and cyber-physical settings also accumulate long-lived sensing histories, so physical safety must be considered together with information security and privacy. Our framework does not solve secure storage, encryption, or access control; rather, the compression/erasure accounting identifies which state summaries must persist, which can be reversibly transported, and which should be deleted.

*Perennial learning*—synonymous with lifelong or continual learning—refers to agentic systems that learn, search, and discover from a stream of data, adapting to new patterns while mitigating catastrophic forgetting [6,7]. Classical continual-learning methods such as elastic weight consolidation, synaptic intelligence, gradient episodic memory, and Progress and Compress focus on retaining useful parameters across task shifts [9,10,11,12]. They are algorithmically successful but mostly agnostic about the thermodynamic cost of a model update, how safe sets evolve during learning, and how the update rule should respect the underlying system structure.

### 1.3. Computational Hardness: Why Perennial Refinement Is Necessary

Most motion-planning problems are PSPACE-hard [1,13,14]. No polynomial-time algorithm can solve the general planning problem exactly. Approximate solutions must therefore be iteratively refined as new observations arrive. The quality of the approximation at any time step depends on the information accumulated thus far—precisely what Kolmogorov complexity measures—while the cost of updating the approximation as the environment changes is precisely what Landauer’s principle quantifies. Navigation under uncertainty exemplifies this: evaluating risk requires simultaneously sensing, identifying, and assessing hazardous objects at multiple spatial scales, from local obstacle avoidance (1:1) to global route planning (1:80,000), each with its own identifiability challenges and constraint geometry [8].

### 1.4. Learning Has Its Own Hamiltonian

The concept of learning Hamiltonians to solve tasks efficiently has deep classical roots in variational calculus [15,16]. The brachistochrone problem—finding the curve z(x) that minimizes descent time—illustrates that nature “learns” optimal trajectories by extremizing an action functional. Our framework makes this literal: a port-Hamiltonian inference engine minimizes a variational free energy (the “action” of inference) subject to dissipative constraints (the “friction” of forgetting). Information thermodynamics interprets prediction and adaptation as irreversibility-budgeted information processing [17,18,19]; PH modeling provides a native decomposition into lossless interconnection, explicit dissipation, and power ports [20]; linearly solvable control, path-integral control, and maximum-entropy RL show that information and control costs share a common variational language [21,22,23].

### 1.5. Contributions

This paper develops a perennial-learning viewpoint in which ideal algorithmic descriptions, practical code proxies, and port-Hamiltonian learning dynamics are kept conceptually distinct. The manuscript now makes that separation explicit.

**Status map**

Formal results: Theorems 1–3 and Propositions 1–5 under the stated assumptions. Design principles: PH channel separation, NCD-gated curriculum scheduling, the practical algorithmic-entropy diagnostic, and the warm-start sequential update rule. Analogies: the Maxwell/Szilard interpretation and Landauer accounting for abstract model updates unless a physical implementation model is specified.

The main technical contributions are as follows:

(i) A clean separation between the ideal Kolmogorov-complexity description of model revision and the practical compressor/MDL surrogate used in optimization, together with a locally scoped calibration statement on the finite serialized model family actually visited by the curriculum.
(ii) A PH “perennial inference engine” architecture that interprets reversible transport, dissipative forgetting, safety barriers, and Casimir locks as separate channels rather than as a single monolithic update.
(iii) A standard passivity/dissipation estimate specialized to curriculum-induced feasible-set contraction, whose novelty lies in its interpretation as a rate limiter for safe curriculum scheduling.
(iv) A local Fisher/Laplace/MDL proxy for posterior codelength contraction and a sequential information-budget proposition that clarifies when warm starts can reduce the effective per-stage sample requirement.
(v) A concrete moving-obstacle double-integrator toy case study with NCD-gated curriculum scheduling, actual simulated trajectories, practical entropy/Fisher/overwrite diagnostics, a passivity-violation stress test, and a compact baseline table.

### 1.6. Roadmap

Section 2 presents the notations, scope, and standing assumptions, restoring the full Kolmogorov-complexity background and stating explicitly where the manuscript uses ideal *K* notions and where it uses the practical proxy $L_{Z}$. Section 3 turns algorithmic update costs into a computable optimal-control surrogate, formalizes the Maxwell/Szilard interpretation as a modeling lens, and develops the information-distance geometry of discovery. Section 4 presents the PH architecture, the toy example, the safety hierarchy, and the passivity speed limit interpreted as a curriculum-admissibility test. Section 5 develops the local Fisher/Laplace codelength proxy, NCD/NID-guided curriculum scheduling, and the sequential information budget. Section 6 specifies a structure-preserving discretization, algorithmic template, and a concrete numerical case study. Section 7 assembles the full engine and states the quantitative headline result together with the broader empirical validation agenda. Section 8 closes with limitations, lifted/reduced PH learning, and open problems.

**Related works**

The present paper is closest in spirit to MDL/Bayesian coding views of learning; information-bottleneck and rate-distortion perspectives on representation change; continual-learning methods such as EWC [9], SI [10], GEM [11], and Progress and Compress [12]; and safe-control/safe-RL methods built around confidence sets, reachability, or barrier certificates. Our point is not to replace these lines of work but to organize them through a PH separation of reversible transport, irreversible overwrite, and safety channels. In control, path-integral control [22] and linearly solvable Markov decision processes [21,23] link information and control costs but do not address lifelong overwrite budgets. Safe RL via Gaussian-process confidence sets [24] and Hamilton–Jacobi reachability [25] handle safety but not the code-length cost of updating safety knowledge. Structure-preserving ML [26,27,28] exploits PH structure for prediction but not for budgeted perennial learning. Geometric optimization and completeness/solvability questions relevant to structured constraints are discussed in [29,30,31].

## 2. Background, Scope, and Standing Assumptions

### 2.1. Description Complexity, Proxies, and Landauer Cost

**Definition** **1** (Prefix Kolmogorov complexity ([32], Ch. 2))**.** *Let U be a universal prefix-free Turing machine. The **prefix Kolmogorov complexity** of a binary string x is*

(1) $$K\left(x\right)\;:=\;\min\left\{\ell (p):U(p)=x\right\},$$

*where ℓ(p) denotes the length of program p.*

**Definition** **2** (Conditional complexity [33])**.** *K(x∣y)=min{ℓ(p):U(p,y)=x}.*

**Definition** **3** (Algorithmic mutual information)**.** *I(x:y)=K(x)−K(x∣y)=K(y)−K(y∣x)+O(log).*

Throughout, O(log) absorbs terms of order O(log(K(x)+K(y))).

For optimization, we use a computable codelength $L_{Z}\left(x\right)$, obtained from a fixed lossless compressor or MDL code [4,34]. Unless otherwise stated, the primary compressor is LZMA/XZ with preset 6 applied to a canonical UTF-8 byte string. Each environment is serialized as sorted-key JSON metadata, followed by ordered, quantized obstacle boundary samples and, for grid tasks, row-major signed-distance samples. The notation $L_{Z}$ is reserved for this practical proxy throughout this paper, and Appendix A provides the serialization, quantization, and sensitivity checks against gzip/zlib level 6 and a parameter-vector encoding. Exact *K* is uncomputable [32]; therefore, any formal theorem about an implemented algorithm must use $L_{Z}$ or another explicit surrogate.

The Shannon entropy of a computable distribution *P* over a countable set X is $H\left(P\right)=\sum_{x}P\left(x\right)\log\left(1/P\left(x\right)\right)$.

**Theorem** **1** (Entropy–complexity bridge ([32], Thm. 8.1.1))**.** *For every recursive distribution P,*

(2) $$0\;\leq \;\sum_{x}P\left(x\right)\;K\left(x\right)-H\left(P\right)\;\leq \;c_{P},$$

*where $c_{P}$ depends only on the length of the shortest program computing P.*

**Remark** **1** (Implication for perennial learning)**.** *A learning agent maintains a model parameterized by θ, inducing a distribution $P_{\theta }$ over observations. The agent’s “understanding” of the data is the extent to which $P_{\theta }$ compresses observations, i.e., the extent to which $\sum P_{\theta }\left(x\right)K\left(x\right)$ approaches $H(P_{\theta })$. Discovery corresponds to finding shorter descriptions: when the agent discovers structure, K(model) may increase modestly but H(data∣model) decreases dramatically, and the net algorithmic entropy decreases.*

**Theorem** **2** (Landauer’s principle [35])**.** *Any logically irreversible device that erases b bits must dissipate at least $Q_{\mathrm{erase}}\geq b\;k_{B}T\ln2$ of heat into a bath at temperature T. Reversible reorganization avoids this lower bound in principle [2,36,37].*

**Remark** **2** (What the Landauer lower bound actually applies to)**.** *Theorem 2 lower-bounds the heat cost of the irreversible part of an update in a concrete physical device. When the manuscript applies Landauer accounting to abstract model updates, it does so as a formal bookkeeping convention for irreversible overwrite unless a computation substrate is explicitly modeled. Thus, $k_{B}T\ln2$ per erased bit is not asserted to be the literal energy paid by a software optimizer. The PH architecture below is designed to separate reversible transport from irreversible overwrite as clearly as possible [38].*

### 2.2. Port-Hamiltonian Systems

**Definition** **4** (Port-Hamiltonian systems [20])**.** *Let $X\subseteq R^{d_{x}}$ and let H:X→R be $C^{1}$. A **port-Hamiltonian system** (PH) has the form*

(3) $$\dot{x}=\left(J(x)-R(x)\right)\nabla H\left(x\right)+G\left(x\right)u\left(t\right),$$

*where $J\left(x\right)=-J{\left(x\right)}^{⊤}$ is the interconnection structure, $R\left(x\right)=R{\left(x\right)}^{⊤}⪰0$ is the dissipation matrix, and G(x) is the input-port map. The power-conjugate output is $y\left(x\right)=G{\left(x\right)}^{⊤}\nabla H\left(x\right)$.*

**Proposition** **1** (Passivity identity [20])**.** *Along any sufficiently smooth trajectory of* (3)*,*

(4) $$\frac{\;d}{\;dt}H\left(x\left(t\right)\right)=-\nabla H{\left(x\left(t\right)\right)}^{⊤}R\left(x\left(t\right)\right)\nabla H\left(x\left(t\right)\right)+y{\left(t\right)}^{⊤}u\left(t\right).$$

*In particular, when u≡0, the Hamiltonian is nonincreasing.*

**Definition** **5** (Casimir lock [20])**.** *A smooth function C:X→R is a **Casimir invariant** if J(x)∇C(x)=0 for all x∈X. Casimir invariants are preserved along every PH trajectory regardless of the choice of H. We use the phrase **Casimir lock** for an engineered invariant that the learner is not allowed to overwrite.*

No theorem in this paper proves that PH is uniquely optimal among all learning architectures. The PH decomposition is used because it is structurally interpretable: reversible interconnection and irreversible dissipation are explicit, and hard invariants can be encoded at the Poisson and dissipative bracket levels [39,40].

### 2.3. Standing Assumptions

**Assumption** **1** (PH regularity and compact sublevels)**.** *The maps H,J,R,G are locally Lipschitz on X, H is $C^{2}$, and all trajectories of interest remain in a compact sublevel set $X_{E_{0}}:=\left\{x\in X:H\left(x\right)\leq E_{0}\right\}$. On each such set,*

(5) $$D_{\max}\left(E_{0}\right):=\underset{x\in X_{E_{0}}}{\sup}\nabla H{\left(x\right)}^{⊤}R\left(x\right)\nabla H\left(x\right)<\infty .$$

**Assumption** **2** (Observation model and local asymptotic normality)**.** *Observations satisfy*

(6) $$y_{i}=h_{\xi^{⋆}}\left(x_{i}\right)+\varepsilon_{i},\;\varepsilon_{i}∼N\left(0,\Gamma_{y}\right),$$

*where $\Gamma_{y}≻0$ and $h_{\xi }\left(x\right)$ is $C^{2}$ in (x,ξ). For the local Fisher proxy, we assume that the posterior around the current iterate is well approximated by a Gaussian with a precision matrix equal to the accumulated Fisher information.*

**Assumption** **3** (Local/probabilistic proxy calibration)**.** *Whenever a theorem transfers an ideal K-based statement to the implemented code, the comparison is restricted to the finite serialized model family actually visited by the curriculum, denoted by $\Xi_{T}$. With probability at least 1−δ over the sampling/serialization process,*

(7) $$|L_{Z}\left(\xi \right)-K\left(\xi \right)|\leq c_{Z}\left(\delta \right),\;\forall \;\xi \in \Xi_{T}.$$

*Outside $\Xi_{T}$, the manuscript makes no global calibration claim.*

**Assumption** **4** (Sequential drift and warm start)**.** *Between curriculum stages t−1 and t, only an active block $S_{t}\subseteq \left\{1,\ldots ,d_{\xi }\right\}$ of size $\Delta k_{t}:=\left|S_{t}\right|$ changes materially, and the restricted Fisher information on that block satisfies $I_{t}{|}_{S_{t}}⪰\lambda_{⋆}I$ for some $\lambda_{⋆}>0$. The estimator is initialized from the previous stage rather than from scratch.*

**Remark** **3** (Scope)**.** *Assumption 3 is deliberately local: it is a statement about the finite family of serialized models actually visited by the curriculum rather than a universal theorem about arbitrary compressors or arbitrary task classes. The main optimization problem and all numerical examples can be read entirely in terms of $L_{Z}$ without appealing to K at all.*

## 3. Thermodynamic Formulation of Perennial Learning

In this section, we first explain how the lifelong regularizer uses a computable code length. Next, we describe how the optimal-control objective is a surrogate for algorithmic rate distortion while introducing an entropy-style discovery diagnostic. Finally, we formalize the Maxwell demon analogy and develop information distance as the geometry of discovery.

### 3.1. Ideal and Practical Lifelong Regularizers

Let $\xi_{t}$ denote the model or solver description at curriculum step *t*.

**Definition** **6** (Ideal and practical overwrite cost)**.** *The ideal bit-overwrite count at step t is $\Delta K_{t}^{⋆}:={\left[K\left(\xi_{t}\right)-K\left(\xi_{t-1}\right)\right]}_{+}$. The practical overwrite count is ${\hat{\Delta K}}_{t}:={\left[L_{Z}\left(\xi_{t}\right)-L_{Z}\left(\xi_{t-1}\right)\right]}_{+}$. The corresponding lifelong penalties are*

(8) $$\Omega_{\mathrm{life}}^{⋆}:=\sum_{t=1}^{T}\Delta K_{t}^{⋆},\;\Omega_{\mathrm{life}}^{Z}:=\sum_{t=1}^{T}{\hat{\Delta K}}_{t}.$$

**Proposition** **2** (Calibration transfer)**.** *Under Assumption 3, $|\Delta K_{t}^{⋆}-{\hat{\Delta K}}_{t}|\leq 2c_{Z}$ for each t, and therefore $|\Omega_{\mathrm{life}}^{⋆}-\Omega_{\mathrm{life}}^{Z}|\leq 2Tc_{Z}$.*

**Proof.**  The map $a\mapsto {\left[a\right]}_{+}$ is 1-Lipschitz, so

$$|\Delta K_{t}^{⋆}-{\hat{\Delta K}}_{t}\left|\;\leq \;\right|\left(K\left(\xi_{t}\right)-K\left(\xi_{t-1}\right)\right)-\left(L_{Z}\left(\xi_{t}\right)-L_{Z}\left(\xi_{t-1}\right)\right)|\;\leq 2c_{Z}.$$

Summing over *t* proves the second bound.    □

### 3.2. From Algorithmic Rate Distortion to a Computable Optimal Control Problem (OCP)

A critical constraint on any learning process is the principle of nonincreasing mutual information.

**Theorem** **3** (Nonincreasing mutual information ([32], Thm. 8.1.4))**.** *For deterministic processing z=f(x),*

(9) I(z;y)≤I(x;y)+K(f)+O(1).

*Data processing cannot create information; any apparent information gain from computation is bounded by the complexity of the computation itself.*

The *algorithmic rate-distortion function* sharpens the compression–fidelity tradeoff.

**Definition** **7** (Algorithmic rate distortion ([32,41], Def. 8.1.5))**.** *For an individual object x and distortion measure d,*

(10) $$r_{x}\left(\delta \right)\;=\;\min_{y}\left\{K(y):d(x,y)\leq \delta \right\}.$$

This provides a per-object compression–fidelity tradeoff rather than a statistical one. The PH solver’s variational free energy (Section 4) instantiates exactly this tradeoff: the agent seeks the shortest model (minK(θ)) whose predictions match the data within tolerance.

**Definition** **8** (Thermodynamic work of model update ([32], Thm. 8.2.4))**.** *The minimal thermodynamic work required to transform model state x to model state y is*

(11) W(y∣x)=K(x)−K(y).

If the new model is simpler (K(y)<K(x)), the system *extracts* work—it has discovered structure. If the new model is more complex (K(y)>K(x)), the system *pays* work—it is storing more information.

**Remark** **4** (Speed–dissipation tradeoff)**.** *The time-bounded complexity satisfies $K^{t}\left(x\right)>K\left(x\right)$ in general. A demon (learning agent) with limited computation time pays $K^{t}\left(x\right)-K\left(x\right)$ in* excess *dissipation. This gap constrains the regret of Differential Policy Optimization (DPO) [42] (Section 5.4) and is consistent with the reversible/adiabatic-computation viewpoint that time and space resources can be traded against energy expenditure [2].*

In optimization, *K* is replaced with its computable surrogate. We therefore define the running cost using observable distortion and practical codelength increments [43].

**Definition** **9** (Computable perennial OCP)**.** *At stage t, with state x, control u, observation $y_{t}$, model $\xi_{t}$, and meta-parameters η (the top-level policy/design variables; see Section 4.1), define*

(12) $$\begin{array}{cc} \ell_{t}\left(x,u;E_{t}\right)\; & =\lambda_{\mathrm{err}}\underset{\mathrm{predictive}\;\mathrm{distortion}}{\underset{︸}{\frac{1}{2}{∥y_{t}-h_{\xi_{t}}\left(x\right)∥}_{\Gamma_{y}^{-1}}^{2}}}+\lambda_{\mathrm{code}}\underset{\mathrm{irreversible}\;\mathrm{update}\;\mathrm{proxy}}{\underset{︸}{{\hat{\Delta K}}_{t}}}\\ \; & \;+\lambda_{\mathrm{safe}}\underset{\mathrm{safety}\;\mathrm{barrier}/\mathrm{margin}}{\underset{︸}{\psi_{F_{t}}\left(x\right)}}+\lambda_{u}{∥u∥}^{2}. \end{array}$$

*The stage objective is*

(13) $$J_{t}\left(\eta \right):=E\;\left[\int_{0}^{T_{t}}\ell_{t}\left(x\left(s\right),u\left(s\right);E_{t}\right)\;\;ds+\phi_{t}\left(x\left(T_{t}\right)\right)\right].$$

### 3.3. Algorithmic Entropy as a Discovery Diagnostic

We now define the algorithmic entropy of physical systems following Li and Vitányi [32], §8.5–8.6 and introduce a computable surrogate for monitoring discovery [44].

**Definition** **10** (Algorithmic entropy ([32], Def. 8.6.2))**.** *The **algorithmic entropy** of a macrostate x is*

(14) $$S_{\;A}\left(x\right)\;=\;\left(k_{B}\ln2\right)\left(K\left(x\right)+H_{x}\right),$$

*where K(x) is the prefix complexity of the macroscopic description and $H_{x}=S_{B}\left(x\right)/\left(k_{B}\ln2\right)$ is the log-volume of the macrostate.*

The decomposition splits total physical entropy into the complexity of what we know (the regularity) and our ignorance about the microstate given the macroscopic description.

**Remark** **5** (Diagnosis: $\;dS_{\;A}/dt$ sign criterion)**.** *For a regular (compressible) microstate, increasing measurements cause $H_{x}$ to decrease rapidly while K(x) increases slowly, so the net $S_{\;A}$ decreases—discovery is occurring. For a random (incompressible) microstate, $H_{x}$ decreases but K(x) increases at the same rate, so $S_{\;A}$ remains flat—the agent is memorizing noise. The sign of $\;dS_{\;A}/dt$ is the algorithmic thermodynamic diagnosis of identifiability: regions where $\;dS_{\;A}/dt<0$ are identifiable; regions where $\;dS_{\;A}/dt\approx 0$ are not.*

**Definition** **11** (Coarse-grained algorithmic entropy ([32], Def. 8.6.6))**.**

(15) $$H_{\mu }^{n}\left(\omega \right)\;=\;\underset{i\leq n}{\inf}\left\{H_{\mu }\left(\Gamma_{\omega_{1:i}}\right)\right\},$$

which satisfies the second law (strong entropy growth) and converges: $H\left(\omega \right)=\lim_{n\to \infty }H^{n}\left(\omega \right)=\inf_{x\in {\left\{0,1\right\}}^{*}}\left\{H\left(\Gamma_{x}\right):\omega \in \Gamma_{x}\right\}$.

The optimal measurement precision $n_{0}$ is the level at which additional complexity $K(\omega_{1:n})$ no longer yields a sufficient decrease in $\log\mu (\Gamma_{\omega_{1:n}})$. This defines a natural stopping criterion for measurement refinement in the curriculum (Section 5.2).

For monitoring purposes, we use a computable surrogate.

**Definition** **12** (Practical algorithmic-entropy surrogate)**.** *Let $m_{t}$ denote the current compressed model description, and let $H_{t}$ denote a coarse uncertainty volume (e.g., log-volume of a posterior credible ellipsoid). Define*

(16) $$\hat{S_{A}}\left(t\right):=\left(k_{B}T\ln2\right)\left(L_{Z}\left(m_{t}\right)+H_{t}\right).$$

**Figure 1:**
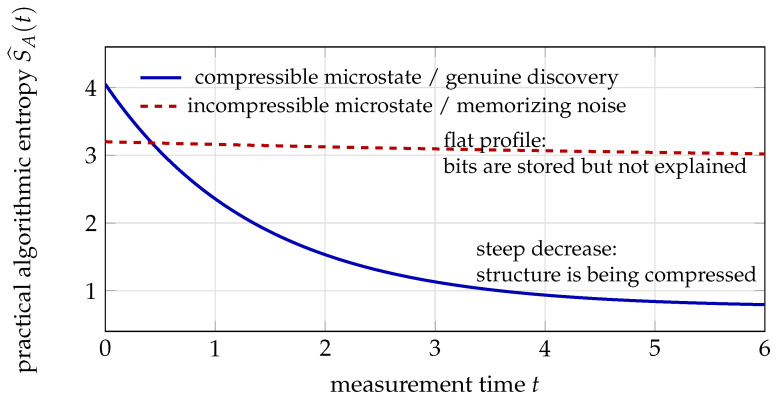
Schematic diagnostic; not empirical data. **Algorithmic-entropy diagnostic (Section 3.3).** Decreasing ${\hat{S}}_{A}\left(t\right)$ indicates that measurements are reducing uncertainty faster than model codelength grows (genuine discovery); a nearly flat curve indicates memorization of noise. The figure is purely schematic; it is intended to visualizes a diagnostic criterion rather than report empirical data.

**Remark** **6** (Status of the entropy diagnostic)**.** *Figure 1 should be read as a design diagnostic rather than as a theorem in which $\hat{S_{A}}\left(t\right)$ is monotone in every learning problem. The operational point is as follows: monitor whether uncertainty is being converted into compression or merely into parameter count.*

### 3.4. Maxwell’s Demon: The Learning Agent as a Thermodynamic Engine

The Maxwell/Szilard discussion below is used as the motivation and as a bookkeeping analogy. The formal claims of this paper are the explicit propositions and assumptions rather than the analogy itself.

The Szilard engine [45] provides the following canonical thought experiment: a demon with memory observes a single-molecule gas, records the molecule’s position, and extracts $k_{B}T\ln2$ of work per observation cycle. The apparent violation of the second law is resolved by Bennett [36]: the demon must *erase* its memory to complete the cycle, incurring a Landauer cost $\geq k_{B}T\ln2$ [46].

**Claim** **1** (Thermodynamic balance sheet ([32], §8.6.1, Claim 8.6.1))**.** *For a combined system (engine + demon) with algorithmic entropy $S_{\;A}=\left(k_{B}\ln2\right)\left(K\left(x\right)+H_{y}\right)$, where K(x) is the complexity of the demon’s memory and $H_{y}$ is the Boltzmann part of the engine, the net heat gained over one cycle satisfies*

(17) $$\Delta Q=\left(S_{A}^{f}-S_{A}^{i}\right)T=\Delta Q^{+}+\Delta Q^{-}\;\leq \;0,$$

*where $\Delta Q^{+}=\left(S_{B}^{f}-S_{B}^{i}\right)T$ is the heat from the engine’s entropy change and $\Delta Q^{-}=\left(K\left(f\right)-K\left(i\right)\right)\cdot k_{B}T\ln2$ is the heat lost to the demon’s memory update.*

One possible mapping to perennial learning is shown in Table 1.

From the demon analysis, the physically correct form of the lifelong regularizer is

(18) $$\Omega_{\mathrm{life}}\left(\eta \right)\;\propto \;\sum_{\mathrm{tasks}}{\left[K\left(\xi_{\mathrm{new}}\right)-K\left(\xi_{\mathrm{old}}\right)\right]}_{+},$$

penalizing cumulative positive complexity increase across task transitions.

**Remark** **7** (Intelligent vs. unintelligent erasure)**.** *An unintelligent demon erasing a random-looking string x pays n bits (the full length). An intelligent demon that recognizes that x encodes a compressible object compresses to K(x)≪n before erasing, paying much less. With limited time, the demon pays $K^{t}\left(x\right)>K\left(x\right)$. This speed–dissipation tradeoff (Remark 4) constrains the DPO regret bound and aligns with the reversible-computation perspective that extra time/space can be exchanged for reduced energetic cost [2].*

### 3.5. Information Distance and the Geometry of Discovery

We develop the information distance as the metric for measuring learning progress and guiding the curriculum, following Li and Vitányi [32], §8.3–8.4. On the computational side, the normalized-compression viewpoint of Li et al. [3] and the clustering-by-compression program of Cilibrasi and Vitányi [5] are central rather than peripheral: they supply the operational surrogate used later for curriculum gating.

**Definition** **13** (Information distance ([32], §8.3))**.** *The **max distance** between strings x and y is*

(19) $$E_{1}\left(x,y\right)\;=\;\max\left\{K(x|y),\;K(y|x)\right\}.$$

*The **sum distance** is*

(20) $$E_{3}\left(x,y\right)\;=\;K\left(x|y\right)+K\left(y|x\right)\;\pm \;O\left(\log\right).$$

The sum distance $E_{3}$ measures the total irreversible bit flow during a reversible computation from *x* to *y* and is the correct cost metric for the PH solver, which consumes observations (bits in) and discards old beliefs (bits out).

**Theorem** **4** (Universality ([32], Thm. 8.3.2))**.** *$E_{1}$ is minimal among all admissible distances: every computable distance between x and y is at least $E_{1}\left(x,y\right)$ up to an additive constant.*

**Definition** **14** (Normalized information distance (NID) ([32], Def. 8.4.1))**.**

(21) $$e\left(x,y\right)\;=\;\frac{\max\{K(x|y),\;K(y|x)\}}{\max\{K(x),\;K(y)\}}.$$

*This takes values in [0,1] and satisfies metric properties ([32], Thm. 8.4.1).*

The practical approximation replaces *K* with the output length of a compressor *Z*, yielding the normalized compression distance (NCD) [3,5]:

(22) $${\mathrm{NCD}}_{Z}\left(x,y\right)\;=\;\frac{Z(xy)-\min\{Z(x),Z(y)\}}{\max\{Z(x),Z(y)\}}.$$

We address how information and compression distances connect to perennial learning:

(a) Task relatedness for curriculum design: $e(E_{t},E_{t+1})$ measures how much the task structure changes between curriculum steps.
(b) Model-change tracking: $e(\xi_{t},\xi_{t+1})$ measures how much the PH solver parameters have changed; a large NID implies a higher Landauer cost.
(c) Discovery progress: $e({\mathrm{model}}_{t},\mathrm{ground}\;\mathrm{truth})$ monotonically decreases, signaling convergence toward the ground truth.
(d) Safe feasibility-set change: $e\left(F\left(\kappa_{t}\right),F\left(\kappa_{t+1}\right)\right)\leq \delta_{\mathrm{safe}}\left(\tau \right)$ bounds the safe curriculum step size, where κ parameterizes the constraint geometry (defined in Example 1) and $\delta_{\mathrm{safe}}$ is the NCD-derived threshold (defined in Section 5.2).

## 4. The Perennial Inference Engine

This section turns the abstract ingredients into a dynamical architecture. The key claim is architectural rather than variational: if observation, reversible transport, dissipation, and hard invariants are kept as separate channels, then update cost and safety become inspectable instead of being hidden inside a generic optimizer.

### 4.1. Architecture

The meta-policy $\pi_{\eta }$ maps an environment E to PH solver parameters $\xi =\pi_{\eta }\left(E\right)$. The (q,p)-form PH solver is

(23) $$\left(\begin{array}{c} \dot{q}\\ \dot{p} \end{array}\right)=\left(\begin{array}{c} \nabla_{p}H_{\xi }\\ -\nabla_{q}H_{\xi } \end{array}\right)+\left(\begin{array}{c} 0\\ -R_{\xi }\nabla_{p}H_{\xi } \end{array}\right)+\left(\begin{array}{c} 0\\ G_{\xi }u_{\xi } \end{array}\right).$$

**Figure 2:**
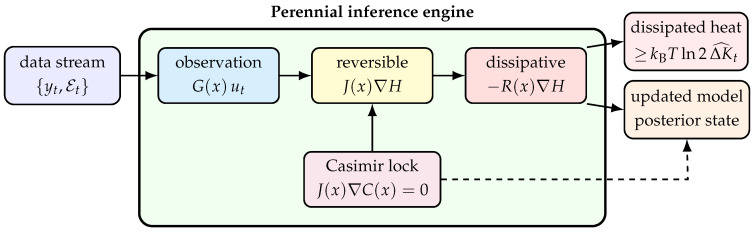
Perennial inference engine architecture display. Three explicit components in differential form handle data input, reversible transport, and irreversible (dissipative) overwrite. Only the dissipative branch carries a Landauer lower bound; the Casimir lock (dashed arrow) enforces the “never overwrite” structural invariant without dissipating energy.

The stochastic extension is

(24) $$\;dx=\left[(J(x)-R(x))\nabla H(x)+G(x)u(t)\right]\;dt+\Sigma \left(x\right)\circ \;dW_{t},$$

where $W_{t}$ is a Wiener process and ∘ denotes the Stratonovich integral [47]. Figure 2 shows the architecture of a perennial inference engine.

Table 2 summarizes the thermodynamic interpretation. The passivity inequality (Proposition 1) ensures that, in the absence of external input, the energy (and hence the “distance to danger”) can only decrease—this is the energy certificate for safety. In the stochastic extension, the passivity inequality would be replaced with weak passivity [48].

The three knowledge regimes introduced by PHAST [49] connect to Kolmogorov cost:

- Known: V(q),M(q),D(q) are given; low K(ξ); Casimir-protectable.
- Partial: some structure is given, some is learned; moderate K(ξ).
- Unknown: everything is learned from data; high K(ξ); full Landauer cost.

The meta-optimization over environments reads

(25) $$\min_{\eta }\;E_{E∼P}\left[J(\eta ;E)\right]+\beta \;\Omega_{\mathrm{struct}}\left(\eta \right)+\gamma \;\Omega_{\mathrm{life}}\left(\eta \right),$$

where the OCP functional is

(26) $$J\left(\eta ;E\right)=E_{x_{0}∼\mu_{E}}\;\left[\int_{0}^{T}\ell \left(x_{E}^{\pi_{\eta }\left(E\right)}\left(t\right),E\right)\;dt+\phi \left(x_{E}^{\pi_{\eta }\left(E\right)}\left(T\right),E\right)\right].$$

### 4.2. Running Toy Example: A Planar Double Integrator with a Moving Obstacle

The running example is a planar double integrator with state x=(q,p), position $q\in R^{2}$, momentum $p\in R^{2}$, and goal $q_{g}\in R^{2}$. At curriculum stage τ, a circular obstacle has center c(τ) and radius r(τ).

**Example** **1** (Moving-obstacle PH controller)**.** *Let*

(27) $$H\left(q,p;\kappa \right)=\frac{1}{2}p^{⊤}M^{-1}p+\frac{\omega_{g}}{2}{∥q-q_{g}∥}^{2}-\alpha \log g_{\varepsilon }\left(q;\kappa \right),$$

*with*

(28) $$g_{\varepsilon }\left(q;\kappa \right):={∥q-c\left(\tau \right)∥}^{2}-r{\left(\tau \right)}^{2}+\varepsilon ,\;\kappa \left(\tau \right):=\left(c\left(\tau \right),r\left(\tau \right)\right).$$

*The barrier is smoothed by ε>0 to avoid singular stiffness. The plant uses the canonical PH structure*

$$J_{\mathrm{plant}}=\left[\begin{array}{cc} 0 & I\\ -I & 0 \end{array}\right],\;R_{\mathrm{plant}}\left(q\right)=\mathrm{diag}\left(0,0,\beta_{\|},\beta_{⊥}\left(q\right)\right),$$

*where $\beta_{⊥}\left(q\right)$ increases near the obstacle in the normal direction. Because the canonical double integrator has no nontrivial Casimirs, the Casimir lock is implemented on the controller side by augmenting the interconnection with a memory coordinate z whose bracket is chosen so that a momentum-budget quantity remains invariant. Hard invariants need not belong to the physical plant alone; they may live in the learning/control interconnection.*

The example is reused in Section 5 and Section 6. The obstacle moves slowly, the barrier reshapes accordingly, and the learner warm starts from the previous stage rather than relearning from scratch.

### 4.3. Safety Hierarchy: Casimir, Barrier, Dissipation

Safety in the perennial-learning context requires three nested guarantees:

(1) Instantaneous safety: x(t)∈F(κ(t)) at every *t*.
(2) Transitional safety: when F contracts, the system reaches the new set without leaving the old one during transition.
(3) Informational safety: the agent’s model of the boundary ∂F has low enough *K* to reliably distinguish safe from unsafe.

**Figure 3:**
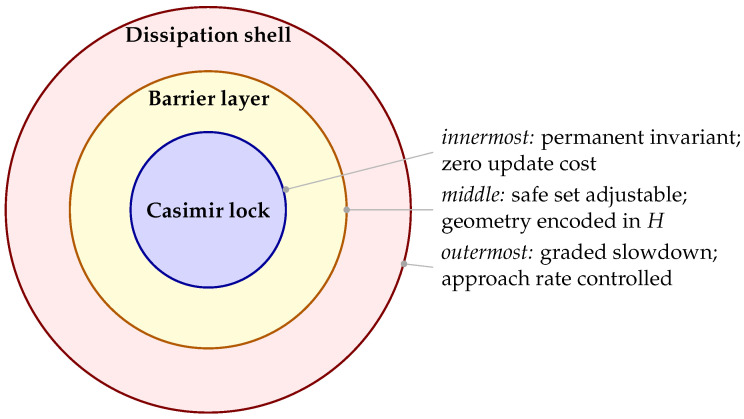
Schematic diagnostic, not empirical data. Safety hierarchy (Section 4.3). Permanent structure belongs in Casimirs, geometric changes belong in barriers, and approach-rate control belongs in dissipation. Combining all three into a single penalty obscures both safety and update cost.

As shown in Figure 3, there are three safety mechanisms that operate at different time scales and with different update costs:

1. Casimir lock: a permanent structural invariant that should never be overwritten during normal operation.
2. Barrier shaping: a tunable potential that changes when the geometry of the safe set changes.
3. Dissipation shaping: a graded slowdown that governs how aggressively the state may approach the boundary.

We list out tradeoffs among three safety mechanisms in Table 3. For the toy example, the stage-wise effective feasible set is

(29) $$F_{\mathrm{eff}}\left(\tau \right):=\left\{x\in X:H\left(x;\kappa \left(\tau \right)\right)\leq E_{\max}\left(\tau \right),\;C_{j}\left(x\right)=c_{j}\;\mathrm{for}\;\mathrm{all}\;\mathrm{locked}\;\mathrm{invariants}\right\}.$$

**Mechanism 1: Dynamic potential shaping**

Decompose the potential as

(30) $$V_{\xi }\left(q;E\right)=V_{\mathrm{task}}\left(q;E\right)+V_{\mathrm{barrier}}\left(q;\kappa \right),$$

where the log-barrier enforces constraint feasibility:

(31) $$V_{\mathrm{barrier}}\left(q;\kappa \right)=-\alpha \sum_{i}\log\left(g_{i}\left(q;\kappa \right)\right).$$

As q→∂F, $V_{\mathrm{barrier}}\to \infty$. Passivity (dH/dt≤0) prevents the trajectory from gaining energy to climb the barrier.

**Mechanism 2: Anisotropic dissipation shaping**

(32) $$R_{\xi }\left(q,p;E\right)=R_{0}\left(E\right)+R_{\mathrm{margin}}\left(q;\kappa \right),$$

where the margin dissipation increases near the boundary:

(33) $$R_{\mathrm{margin}}\left(q;\kappa \right)=\beta \sum_{i}\frac{1}{g_{i}{\left(q;\kappa \right)}^{2}}\;\nabla g_{i}\;\nabla g_{i}^{\;⊤}.$$

This is *anisotropic*: strong damping normal to the constraint surface and weak damping tangentially. The trajectory can explore along the boundary (learning its geometry for identifiability) while being strongly damped against crossing it.

**Mechanism 3: Casimir invariants as hard safety constraints**

Casimir functions C(x) satisfying $\{C,H_{\xi }\}=0$ (Definition 5) are preserved for *all* Hamiltonians, surviving potential reshaping, dissipation adjustment, and curriculum changes. From a Kolmogorov perspective, Casimirs incur zero marginal Landauer cost: once encoded in the Poisson-bracket structure, they require no ongoing memory, measurement, or erasure.

**Remark** **8** (Informational cost of safety)**.** *Updating the agent’s model of what is safe carries an overwrite cost. In a physical implementation, the irreversible component is lower-bounded by $\Delta Q_{\mathrm{safety}}\geq k_{B}T\ln2\cdot K\left(F\left(\kappa_{t+1}\right)|F\left(\kappa_{t}\right)\right)$ for the erased portion of the description. For abstract software updates, this expression should be read as a formal overwrite budget rather than as a literal device-level energy audit.*

### 4.4. Passivity Speed Limit for Constraint Tightening

The next proposition is a standard passivity/dissipation estimate tailored to curriculum-induced feasible-set contraction. Its novelty here does not lie in the inequality itself but in the interpretation of $D_{\max}$ as a rate limiter for safe curriculum scheduling.

**Proposition** **3** (Passivity speed limit)**.** *Under Assumption 1, consider the trajectory in (3) satisfying the non-energy-extracting condition*

(34) $$y{\left(t\right)}^{⊤}u\left(t\right)\geq 0\;\mathrm{for}\;\mathrm{almost}\;\mathrm{every}\;t\in \left[0,T\right].$$

*Suppose $x\left(0\right)\in X_{E_{0}}$ with $H\left(x\left(0\right)\right)=E_{0}$, and any transition into the updated feasible set requires the energy to reach some level at most $E_{1}<E_{0}$ while the trajectory remains inside $X_{E_{0}}$. Then, every such transition satisfies*

(35) $$T\;\geq \;\frac{E_{0}-E_{1}}{D_{\max}\left(E_{0}\right)}.$$

*The curriculum cannot tighten constraints faster than the system can dissipate energy.*

**Proof.**  By Proposition 1,

$$\dot{H}\left(x\left(t\right)\right)=-\nabla H^{⊤}R\nabla H+y^{⊤}u\geq -D_{\max}\left(E_{0}\right),$$

where the inequality uses (34) and $D_{\max}\left(E_{0}\right)$. Integrating from 0 to *T*, $E_{1}-E_{0}\geq -D_{\max}\left(E_{0}\right)T$, which rearranges to (35).    □

**Remark** **9** (Interpretation and limits)**.** *Proposition 3 is a* worst-case *lower bound. If an external controller is allowed to extract energy ($y^{⊤}u<0$), if $X_{E_{0}}$ is not compact, if $D_{\max}\left(E_{0}\right)$ is not finite, or if stochastic excitation/exploration policies inject net power, the proposition no longer applies in the stated form. Section 6.4 gives a numerical stress test: the direct A→C obstacle jump has ${\mathrm{NCD}}_{Z}=0.265>\delta_{\mathrm{safe}}=0.245$ and, when forced with a lagged barrier, produces negative clearance, whereas the admitted NCD-gated chain maintains positive clearance.*

### 4.5. Coverage, Identifiability, and Entropy Reduction Rate

The occupation measure $\rho_{\eta ,E}=\frac{1}{T}\int_{0}^{T}\delta_{z(t)}\;dt$ determines which microstates have their K(x) reduced by observation. The coverage discrepancy is

(36) $$\mathrm{Cov}\left(\eta ;E\right)=D\left(\rho_{\eta ,E},\;\nu_{E}\right),$$

where the target measure $\nu_{E}$ weights regions of high $S_{\;A}$ (i.e., regions with much to discover), where $-\;dS_{\;A}/\;dt$ is achievable (i.e., where the microstate is compressible rather than random).

The Fisher/observability proxy for algorithmic-entropy reduction is (using the same symbol conventions as Section 5.1)

(37) $$I_{T}\left(\xi \right):=\int_{0}^{T}{\left(\partial_{\xi }h_{\xi }\left(x\left(t\right)\right)\right)}^{\;⊤}\Gamma_{y}^{-1}\left(\partial_{\xi }h_{\xi }\left(x\left(t\right)\right)\right)\;dt.$$

**Claim** **2** (Fisher proxy for local posterior contraction)**.** *Let $\lambda_{\min}\left(I_{T}\left(\xi \right)\right)$ denote the smallest eigenvalue of the accumulated Fisher information matrix defined in* (37)*. Large $\lambda_{\min}\left(I_{T}\left(\xi \right)\right)$ suggests that observations are informative in the weakest identified direction and therefore that the* local *posterior codelength can contract efficiently along the trajectory.*

Note that Claim 2 is a local design claim, not a theorem about exact algorithmic entropy $S_{\;A}$. Proposition 4 (Section 5.1) gives the qualified Laplace/MDL version under Assumption 2.

**Remark** **10** (Safety–identifiability alignment)**.** *Near constraint boundaries, dynamics are typically more structured (the boundary imposes regularity), so $-\;dS_{\;A}/dt$ is higher near boundaries than in the interior. The most informative regions for identifiability are thus exactly where the agent most needs to learn constraint geometry. The spatio-temporal dissipation shaping* (33) *ensures a slow, safe approach. This alignment is task-dependent rather than universal (see Remark 12).*

## 5. Identifiability, Curriculum, and Sequential Complexity

This section focuses on the Fisher-information claim and the measurement-complexity claim.

### 5.1. Fisher Information as a Local Codelength Proxy

For a trajectory x(·) and using the observation model from Assumption 2, the accumulated Fisher information is

(38) $$I_{T}\left(\xi \right):=\int_{0}^{T}{\left(\partial_{\xi }h_{\xi }\left(x\left(t\right)\right)\right)}^{⊤}\Gamma_{y}^{-1}\left(\partial_{\xi }h_{\xi }\left(x\left(t\right)\right)\right)\;dt.$$

To avoid singularities, we work with the ridge-stabilized matrix $I_{T}^{\rho }:=I_{T}+\rho I$ for any ρ>0.

**Proposition** **4** (Local Fisher proxy for posterior codelength contraction)**.** *Under Assumption 2, let the local negative log posterior around the current iterate be approximated by*

$$-\log p\left(\xi |{\mathrm{data}}_{1:T}\right)=\mathrm{const}+\frac{1}{2}{\left(\xi -{\hat{\xi }}_{T}\right)}^{⊤}I_{T}^{\rho }\left(\xi -{\hat{\xi }}_{T}\right)+o(∥\xi -{\hat{\xi }}_{T}{∥}^{2}).$$

*Define the local uncertainty-codelength score $U_{T}^{\rho }:=\frac{1}{2}\log\det\left(I_{T}^{\rho }\right)$. Then,*

(39) $$\frac{d_{\xi }}{2}\log\;\left(\lambda_{\min}\left(I_{T}\right)+\rho \right)\leq U_{T}^{\rho }\leq \frac{d_{\xi }}{2}\log\;\left(\lambda_{\max}\left(I_{T}\right)+\rho \right).$$

*Therefore, maximizing $\lambda_{\min}\left(I_{T}\right)$ increases a worst-direction lower bound on local posterior codelength contraction.*

**Proof.**  Let $\lambda_{1},\ldots ,\lambda_{d_{\xi }}$ be the eigenvalues of $I_{T}$. Then, $U_{T}^{\rho }=\frac{1}{2}\sum_{i}\log\left(\lambda_{i}+\rho \right)$. Since $\lambda_{\min}\leq \lambda_{i}\leq \lambda_{\max}$,

$$\frac{d_{\xi }}{2}\log\left(\lambda_{\min}+\rho \right)\leq U_{T}^{\rho }\leq \frac{d_{\xi }}{2}\log\left(\lambda_{\max}+\rho \right).$$

   □

**Remark** **11** (Status of the Fisher proxy)**.** *Proposition 4 is* not *a theorem about exact algorithmic entropy $S_{\;A}$ or exact Kolmogorov complexity. Under a Laplace approximation, the posterior codelength contains a curvature term $\frac{1}{2}\log\det\left(I_{T}^{\rho }\right)$ plus lower-order constants, so Fisher information is used only as a local statistical codelength proxy. It can fail for multimodal posteriors, sensor aliasing, unobserved boundary parameters, severe model misspecification, or directions in which curvature is large but operationally irrelevant. In the scheduler, it is therefore paired with residual checks and NCD change rather than used as a standalone certificate.*

**Assumption** **5** (Boundary-informative sensing)**.** *There exists a neighborhood $N_{r}\left(\partial F\right)$ of the constraint boundary and a constant $\sigma_{b}>0$ such that the singular values of the Jacobian directions associated with the boundary parameters satisfy*

$$\sigma_{\min}\left(\partial_{\xi_{\partial }}h_{\xi }\left(x\right)\right)\geq \sigma_{b}\;for\;all\;x\in N_{r}\left(\partial F\right).$$

**Remark** **12** (When safety and identifiability align)**.** *Under Assumption 5, dwelling near the boundary can improve $\lambda_{\min}\left(I_{T}\right)$ precisely in the directions that define the safe-set geometry. Without this assumption, the alignment can fail; there are perfectly safe problems whose boundaries are flat, hidden, or sensor-poor. Safety identifiability alignment is task-dependent rather than universal.*

### 5.2. Curriculum Scheduling via NCD and NID

**Notations used in this section**

- $F_{\mathrm{eff}}\left(\tau \right)$: the effective feasible set at curriculum time τ, defined in (29). Always written with the time argument; bare F(κ) refers to the abstract set parameterized by κ, and $F^{t}$ (without subscript eff) is not used.
- $\delta_{\mathrm{safe}}\left(\tau \right)\in \left[0,1\right]$: the admissible NCD threshold at step τ, derived from the Landauer budget $B_{\tau }$ and defined in (42).
- $L_{Z}\left(y|x\right):=L_{Z}\left(xy\right)-L_{Z}\left(x\right)$: practical conditional code length (not the algorithmic conditional K(y∣x)); defined precisely in (40).
- $\Delta_{t}$: total information change in bits at stage *t* (Proposition 5).
- $\Delta k_{t}$: number of materially changed model coordinates at stage *t* (Assumption 4). These two quantities are distinct: $\Delta_{t}=O\left(\Delta k_{t}\log d_{\xi }/\lambda_{⋆}\right)$ under the warm-start assumptions.

**Implementation and sensitivity.**

We compute $L_{Z}$ with a fixed, lossless compressor *Z*. Unless otherwise stated, *Z* is LZMA/XZ with preset 6 applied to a canonical byte string. Each environment is serialized as sorted-key JSON metadata, followed by ordered, quantized obstacle-boundary samples and, for grid tasks, row-major signed-distance samples. All strings are encoded as UTF-8, and all floating-point parameters are quantized before serialization. The practical rule uses

$${\mathrm{NCD}}_{Z}\left(x,y\right)=\frac{Z(xy)-\min\{Z(x),Z(y)\}}{\max\{Z(x),Z(y)\}},$$

and Appendix A reports sensitivity to gzip/zlib level 6 and to a parameter-vector encoding.

**The core problem**

A perennial learner does not encounter all scenarios at once; it moves through them in sequence. The order and pace of that sequence is the curriculum. A poorly ordered curriculum is susceptible to failure in two distinct ways. First, if consecutive tasks are too similar, the agent wastes update budget re-encoding information it already holds. Second, if they are too different, the agent cannot absorb the changes within one dissipation interval—the feasible set $F_{\mathrm{eff}}$ would need to jump discontinuously, which the passivity speed limit (Proposition 3) forbids. Good curriculum design lives in the space between these two failure modes.

**Compression distance as a curriculum metric**

The key insight is that two tasks are informationally close if knowing one makes the other easy to describe. This is precisely what the normalized compression distance ${\mathrm{NCD}}_{Z}$ (Definition 14, (22)) captures, in the spirit of the similarity-metric and clustering-by-compression program of Li et al. [3], Cilibrasi and Vitányi [5]: if the concatenation $Z(E_{t}E_{t+1})$ is barely larger than $Z(E_{t})$ alone, then $E_{t+1}$ is almost entirely predictable from $E_{t}$—the transition carries little new information and is safe to absorb. Conversely, a large ${\mathrm{NCD}}_{Z}$ signals that the new task contains a structure the model has never seen, and the agent must allocate additional update budget before accepting the step.

This compression-based view has the following suggestive thermodynamic reading. Define the practical conditional code length

(40) $$L_{Z}\left(y|x\right)\;:=\;L_{Z}\left(xy\right)-L_{Z}\left(x\right),$$

the extra bits needed to describe *y* given *x* as a side-channel. On the visited serialized family from Assumption 3, $L_{Z}\left(E_{t+1}|E_{t}\right)$ locally approximates $K(E_{t+1}|E_{t})$, so it serves as a practical overwrite proxy for the curriculum step. This does not make ${\mathrm{NCD}}_{Z}$ a universal learning-theoretic metric: it remains compressor- and representation-dependent and must be validated empirically against the transfer cost or safety violations in the task family.

**The budget constraint**

Let $\delta_{\mathrm{safe}}\left(\tau \right)$ denote the fraction of the current code budget available in one curriculum interval, derived from the dissipation capacity $D_{\max}\left(E_{0}\right)$ and the Landauer rate via Proposition 3. A curriculum step is admissible only if its information content fits within that budget. The ideal (uncomputable) condition uses the NID:

(41) $$e\left(F\left(\kappa_{\tau }\right),\;F\left(\kappa_{\tau +1}\right)\right)\;\leq \;\delta_{\mathrm{safe}}\left(\tau \right),$$

and the practical condition replaces the NID with its computable ${\mathrm{NCD}}_{Z}$ surrogate (justified by Assumption 3):

(42) $${\mathrm{NCD}}_{Z}\left(E_{t},E_{t+1}\right)\;\leq \;\delta_{\mathrm{safe}}\left(t\right).$$

Concretely, if the dissipation integral over one step can absorb at most $B_{t}$ bits of erasure work, then $\delta_{\mathrm{safe}}\left(t\right)=B_{t}/Z\left(E_{t}\right)$, so the threshold shrinks as tasks grow more complex and grows as the agent builds dissipative capacity. A step that fails the screen is not forbidden; the scheduler instead inserts intermediate environments until the chain of steps is individually admissible.

**Example** **2** (NCD screening on the moving-obstacle curriculum)**.** *Encode each environment as a structured description (dynamics, goal, sensor model, obstacle parameters). The compressed length Z(E) is a proxy for the model’s minimum description length under that scenario. For a nearby transition $E_{A}\to E_{B}$, where the obstacle moves by two grid cells,*

$$Z\left(E_{A}\right)=222,\;Z\left(E_{B}\right)=222,\;Z\left(E_{A}E_{B}\right)=232,$$

*so ${\mathrm{NCD}}_{Z}\left(E_{A},E_{B}\right)=\left(232-222\right)/222\approx 0.045$. The small numerator (10 bytes) reflects the fact that once $E_{A}$ is known, $E_{B}$ requires almost no additional information to specify—only the new obstacle center.*

*A more disruptive change $E_{A}\to E_{C}$ (obstacle doubles in size and relocates) yields $Z(E_{C})=221$, $Z(E_{A}E_{C})=247$, so ${\mathrm{NCD}}_{Z}\left(E_{A},E_{C}\right)\approx 0.117$. Here, the numerator (25 bytes) reflects that $E_{C}$ contains substantial new geometry the compressor cannot predict from $E_{A}$.*

*If the per-step Landauer budget corresponds to* 18 *new compressed bytes, then $\delta_{\mathrm{safe}}=18/222\approx 0.081$. The first transition (0.045<0.081) is admitted; the second (0.117>0.081) is not, and the scheduler must find an intermediate scene $E_{A^{\prime }}$ with ${\mathrm{NCD}}_{Z}\left(E_{A},E_{A^{\prime }}\right)\leq 0.081$ and ${\mathrm{NCD}}_{Z}\left(E_{A^{\prime }},E_{C}\right)\leq 0.081$ before the full jump is attempted.*

**Curriculum Lifecycle**

**Figure 4:**
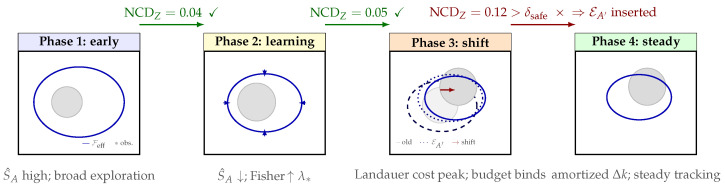
Schematic diagnostic of curriculum lifecycle discussed in Section 5.2. Each panel shows $F_{\mathrm{eff}}\left(\tau \right)$ (solid blue ellipse) and the obstacle (gray disc). Transition arrows above the panels: green = step admitted (${\mathrm{NCD}}_{Z}\leq \delta_{\mathrm{safe}}$); red × = screened, intermediate $E_{A^{\prime }}$ inserted. Inward arrows in Phase 2 show $F_{\mathrm{eff}}$ contracting as the boundary geometry is learned. In Phase 3, the dashed ellipse is the ghost of the old $F_{\mathrm{eff}}$; the dotted ellipse is $E_{A^{\prime }}$; and the red arrow is the obstacle shift. Cost annotations below each panel indicate the dominant information-theoretic regime. Key invariant: the solid blue ellipse deforms continuously—it never jumps.

The four phases shown in Figure 4 describe not just geometric states but distinct information-theoretic regimes, each with its own dominant cost and its own trigger for the transition to the next phase.

1. Early phase (Phase 1 in Figure 4). The agent has limited knowledge of obstacle geometry, so it maintains a large, conservative $F_{\mathrm{eff}}$ that includes a generous safety margin. The feasibility set is over-sized: the agent trades performance for safety by staying well away from the true boundary. Algorithmically, entropy ${\hat{S}}_{A}$ is high (many microstates consistent with the agent’s current model), and  ${\mathrm{NCD}}_{Z}$ between consecutive observations is small because nothing surprising is happening. Transition trigger: once the Fisher information $\lambda_{\min}\left(I_{T}^{\rho }\right)$ rises above $\lambda_{*}$ (Assumption 4), the boundary geometry is sufficiently identified and Phase 2 begins.
2. Learning phase (Phase 2). With reliable boundary estimates, the agent begins tightening $F_{\mathrm{eff}}$—increasing barrier steepness (the scale α in the log-barrier potential (31)) and raising boundary dissipation—so that $F_{\mathrm{eff}}$ contracts toward the true obstacle contour. Each contraction step satisfies (42): the model description shortens as uncertainty collapses, so ${\hat{S}}_{A}$ falls. The Landauer cost per step is low because the agent is mostly deleting uncertainty (erasing microstates it has ruled out) rather than encoding a genuinely new structure. Transition trigger: ${\hat{S}}_{A}$ stops decreasing, signaling that further tightening requires new observations rather than inference.
3. Constraint shift (Phase 3). An external event changes the true constraint—in the double-integrator example, the obstacle moves (red arrow in Figure 4). This injects new information: the old model of obstacle geometry is partially wrong, so ${\mathrm{NCD}}_{Z}\left(E_{t},E_{t+1}\right)$ spikes. The scheduler checks (42): if the spike exceeds $\delta_{\mathrm{safe}}$, the transition is broken into smaller steps. Even so, the agent must pay the Landauer cost ${\hat{\Delta K}}_{t}\approx L_{Z}\left(F_{\mathrm{eff}}\left(t+1\right)|F_{\mathrm{eff}}\left(t\right)\right)$ (using the conditional code notation (40)) for each bit of old geometry it overwrites, and the rate of deformation of $F_{\mathrm{eff}}$ is bounded by Proposition 3. This is the phase in which the thermodynamic cost is highest, and the safety of curriculum ordering matters most.
4. Steady state (Phase 4). The agent has re-adapted to the shifted obstacle and now tracks slow constraint drift with the amortized cost $n_{\mathrm{seq}}=\tilde{O}\left(\Delta k_{t}/\lambda_{⋆}\right)$ measurements per update (Corollary 1), conditional on the warm-start assumptions of Proposition 5, where $\Delta k_{t}$ is the number of parameters that have changed. The Casimir lock ensures that the invariants established in Phase 2 (momentum-budget conservation, obstacle-clearance structure) are not inadvertently erased during re-adaptation. $F_{\mathrm{eff}}$ oscillates gently around the moving boundary rather than making discrete jumps. Return trigger: if ${\mathrm{NCD}}_{Z}$ spikes again above $\delta_{\mathrm{safe}}$, the lifecycle re-enters Phase 3.

**Remark** **13** (Why order matters even when all tasks are eventually visited)**.** *A common objection is that if the agent will eventually see all environments anyway, curriculum ordering is merely a performance optimization. This misses the thermodynamic point: a badly ordered curriculum forces the agent into Phase 3 prematurely and repeatedly, incurring Landauer costs proportional to the* total variation *in the constraint trajectory rather than its net displacement. A well-ordered curriculum—one in which ${\mathrm{NCD}}_{Z}$ between consecutive steps stays below $\delta_{\mathrm{safe}}$—keeps the agent near Phase 2 or Phase 4, where the erasure cost is amortized over many steps rather than concentrated in sudden rewrites [50].*

### 5.3. Sequential Information Budget

**Proposition** **5** (Sequential information budget)**.** *Let $\Delta_{t}$ denote the conditional description change that must be learned at stage t, measured in bits by a practical code such as $L_{Z}\left(\xi_{t}|\xi_{t-1}\right)$. Suppose each newly acquired measurement contributes at most $b_{\mathrm{eff}}$ bits of conditional mutual information about the changed component $I\left(\xi_{t};Y_{i}|\xi_{t-1},H_{t-1}\right)\leq b_{\mathrm{eff}}$ for each i. Then any sequential learner that resolves the changed description must use at least*

(43) $$n_{t}\;\geq \;\frac{\Delta_{t}}{b_{\mathrm{eff}}}.$$

*If, in addition, Assumption 4 holds and the estimator is restricted to the active block $S_{t}$, then a warm-start local estimator can achieve*

(44) $$n_{t}=O\;\left(\frac{\Delta k_{t}\log d_{\xi }+\log\left(1/\delta \right)}{\lambda_{⋆}}\right)$$

*for confidence level 1−δ.*

**Proof.**  The lower bound follows from the following chain rule: $I\left(\xi_{t};Y_{1:n_{t}}|\xi_{t-1},H_{t-1}\right)\leq n_{t}b_{\mathrm{eff}}$. To resolve $\Delta_{t}$ bits of changed description, the left-hand side must be at least $\Delta_{t}$, yielding (43).

Relationship between $\Delta_{t}$ and $\Delta k_{t}$. $\Delta_{t}$ is the total information content in bits that must be acquired; $\Delta k_{t}$ is the number of model coordinates that have changed. Under the warm-start sparsity structure of Assumption 4, each of the $\Delta k_{t}$ changed coordinates contributes at most $O(\log d_{\xi }/\lambda_{⋆})$ bits of local identification cost, so $\Delta_{t}=O\left(\Delta k_{t}\log d_{\xi }/\lambda_{⋆}\right)$, and the lower bound (43) is matched (up to constants) by the upper bound (44). The upper bound is the standard local-identification scaling on the active block under the Fisher lower bound $\lambda_{⋆}$; only $\Delta k_{t}$ coordinates need to be relearned.    □

**Corollary** **1** (Comparison with a cubic cold-start baseline)**.** *Suppose a chosen cold-start identification pipeline obeys $N_{\mathrm{static}}\left(k,d_{\xi }\right)=\Theta \left(k^{3}\log d_{\xi }\right)$. Under the assumptions of Proposition 5, the warm-start sequential stage complexity obeys $N_{\mathrm{seq}}\left(t\right)=O\left(\Delta k_{t}\log d_{\xi }/\lambda_{⋆}\right)$. Hence, the “$k^{3}\to \Delta k$” improvement is valid only relative to that cold-start baseline and only when $\Delta k_{t}\ll k_{t}^{3}$.*

**Example** **3** (Numerical instance of the conditional improvement)**.** *Take $d_{\xi }=256$, cold-start active complexity $k_{t}=8$, changed block size $\Delta k_{t}=2$, and Fisher lower bound $\lambda_{⋆}=0.9$. A cold-start method has $N_{\mathrm{static}}=0.5\;k_{t}^{3}\log d_{\xi }\approx 1419$. A warm-start local estimator with $N_{\mathrm{seq}}\approx 4\Delta k_{t}\log d_{\xi }/\lambda_{⋆}$ requires only about* 49 *measurements. If the drift rises to $\Delta k_{t}=6$, the same formula yields about* 148 *measurements: the budget grows with drift rather than with total task size.*

### 5.4. Where Differential Policy Optimization (DPO) Fits

Differential Policy Optimization (DPO) is an external transport-based optimal control solver whose convergence and regret analysis are obtained from [42,51]. Its attraction here is conceptual: pointwise transport updates align naturally with the reversible branch of the PH decomposition and with Pontryagin-style trajectory optimization [52]. This manuscript does *not* prove that DPO is the unique optimal steering law for the PH learner, nor does it rederive the external $O(K^{5/6})$ regret bound.

**Theorem** **5** (DPO pointwise convergence [42])**.** Let $G_{\theta_{k}}^{\left(j\right)}$ denote the learned transport operator at stage j and step k, and let $G^{*(j)}$ denote the optimal operator. Then,

(45) $$E∥G_{\theta_{k}}^{\left(j\right)}\left(X\right)-G^{*(j)}\left(X\right)∥\;<\;j\;L^{j}\;\frac{\epsilon }{L-1},$$

*where L is the Lipschitz constant and ϵ is the per-stage error.*

**Thermodynamic interpretation of the regret bound**

The regret decomposes into:

- Inference regret: excess cost from suboptimal transport, i.e., $K^{t}\left(x\right)-K\left(x\right)$ from the speed–dissipation tradeoff (Remark 4).
- Safety regret: excess cost from unnecessary conservatism (staying too far from boundaries before learning constraint geometry).

As K→∞, both components decrease. Total excess Landauer dissipation per episode: $O(K^{5/6})$.

**Stochastic extension**

The rough-path extension of DPO [51] handles the noise port $\Sigma \circ \;\;dW_{t}$ in the stochastic PH system (24).

**State-constrained DPO for safety**

Standard DPO applies Pontryagin without state constraints [53]. For safe perennial learning, the constraint $x\left(t\right)\in F_{\mathrm{eff}}\left(\tau \right)$ is incorporated via the PH barrier potential $V_{\mathrm{barrier}}$ (31) and boundary dissipation $R_{\mathrm{margin}}$ (33), converting the constrained problem into an unconstrained one with modified Hamiltonians. The co-state (adjoint) variable becomes the shadow price of constraint proximity.

**Bilevel optimization**

The coupled bilevel optimization governing the curriculum reads

(46) $$\begin{array}{cc} \min_{\eta }\; & \;E_{\tau }\left[J(\eta ;E(\tau ))\right]\;s.t.\;\kappa \left(\tau \right)=C_{\psi }\left(H_{\tau }\right),\;x\left(t\right)\in F_{\mathrm{eff}}\left(\tau \right)\;\forall t,\\ \max_{\psi }\; & \;E_{\tau }\;\left[\lambda_{\min}\left(I_{T}\left(\xi ;E\left(\tau \right)\right)\right)-\alpha \;\mathrm{Cov}\left(\eta ;E\left(\tau \right)\right)\right]\;s.t.\;\dot{\kappa }\left(\tau \right)\leq \kappa_{\max}\left(\tau \right), \end{array}$$

where $\dot{\kappa }\left(\tau \right)\leq \kappa_{\max}\left(\tau \right)$ is the passivity-derived speed limit (Proposition 3). Here, ψ denotes the curriculum-design parameters (the outer optimization variable governing how the curriculum sequence is shaped). This is distinct from $\psi_{F_{t}}\left(x\right)$, which is the scalar safety-barrier/margin term appearing in the running cost (12); the two ψ-objects share a letter but play different roles and are always distinguished by their arguments.

**Remark** **14.** *[Algorithmic temperature and curriculum difficulty] Following Baez and Stay [54], the “algorithmic temperature” is the cost of doubling the number of programs. In the curriculum context, this maps to scenario difficulty: “hotter” scenarios (more stochastic, wider constraint sets) require more erasure work to compress. The curriculum implicitly schedules the temperature of the environments the demon faces [41].*

## 6. Numerical Realization

The theoretical statements above are continuous-time. An implementation needs a discretization that does not destroy the passivity structure the theory relies on.

### 6.1. Discretization, Passivity Margin, and Stiffness Handling

We recommend a splitting integrator with three substeps:

1. Conservative step. Integrate $\dot{x}=J\left(x\right)\nabla H\left(x\right)$ using a symplectic method such as Störmer–Verlet or symplectic Euler [55].
2. Dissipative/barrier step. Integrate $\dot{x}=-R\left(x\right)\nabla H\left(x\right)+G\left(x\right)u$ with a discrete-gradient or average-vector-field method [56], so that a discrete passivity inequality is preserved at the substep level.
3. Stochastic step. Integrate the Stratonovich noise using a midpoint/Heun-type method compatible with the chain rule [47].

For the dissipative substep, a discrete-gradient update yields

(47) $$H\left(x_{n+1}\right)-H\left(x_{n}\right)\leq h\;y_{n}^{⊤}u_{n}-h\;\bar{\nabla }H^{⊤}R\left({\bar{x}}_{n}\right)\bar{\nabla }H,$$

where $\bar{\nabla }H$ is a discrete gradient and *h* is the time step. A Strang split preserves this inequality up to $O(h^{3})$ local and $O(h^{2})$ global error when the subflows are solved to matching order [49].

**Remark** **15** (Barrier stiffness)**.** *The ideal log-barrier −αlogg(q;κ) becomes stiff as g↓0. In numerics, we use the smoothed barrier −αlog(g+ε) from Example 1, together with adaptive stepping or an implicit dissipative substep. The theory remains stated for the ideal barrier; the implementation uses its regularized counterpart.*

**Remark** **16** (Why Stratonovich rather than Itô?)**.** *The Stratonovich formulation is adopted because it obeys the ordinary chain rule and respects coordinate changes on geometric state spaces, which is natural for PH systems. If one rewrites in Itô form, an additional drift correction $\frac{1}{2}\sum_{j}\left(\partial_{x}\Sigma_{\cdot j}\right)\Sigma_{\cdot j}$ appears [57]. Stratonovich models the continuous-time limit of rapidly varying physical perturbations more faithfully when PH geometry matters.*

### 6.2. Algorithmic Template

We provide Algorithm 1 as a general perennial PH learning algorithm under budgeted curriculum updates. The policy update is realized by a time integration step rather than a transition function and NCD is used to compute the budget and guide the learned model sequence do adapt or maintain its current state.
**Algorithm 1** Perennial PH learning with code-budgeted curriculum updates**Input:** prior model $\xi_{0}$, initial environment encoding $E_{0}$, code proxy $L_{Z}$, dissipation budget schedule**for** t=1,2,…,T**do**    encode proposed next environment $E_{t}$ and compute ${\mathrm{NCD}}_{Z}\left(E_{t-1},E_{t}\right)$    **if** ${\mathrm{NCD}}_{Z}\left(E_{t-1},E_{t}\right)>\delta_{\mathrm{safe}}\left(t\right)$ **then**        replace $E_{t}$ by an intermediate environment satisfying the budget    **end if**    warm-start the solver/policy from $\xi_{t-1}$ (DPO or another local transport solver)    update barrier and dissipation parameters for $F_{\mathrm{eff}}\left(t\right)$    integrate the PH dynamics by conservative/dissipative/stochastic splitting    collect new measurements and update $\xi_{t}$    record overwrite proxy ${\hat{\Delta K}}_{t}={\left[L_{Z}\left(\xi_{t}\right)-L_{Z}\left(\xi_{t-1}\right)\right]}_{+}$    monitor $\hat{S_{\;A}}\left(t\right)$, $\lambda_{\min}\left(I_{t}\right)$, and constraint-margin statistics**end for****Output:** updated model sequence ${\left\{\xi_{t}\right\}}_{t=0}^{T}$, safety and code-budget logs

### 6.3. Validation Protocol

**Constrained autonomy**

The moving-obstacle double-integrator example tests whether the PH controller can reshape barriers, preserve controller-side invariants, and reuse previous information safely. Relevant metrics: violation rate, minimum distance to the obstacle boundary, energy ratio $H/E_{\max}$, and drift of locked invariants.

**Sequential data assimilation**

The same framework applies when the “safe set” is a confidence region induced by sensors and physics constraints. Sensor dropout, new sensors, or changing observation operators generate the sequential-drift setting from Assumption 4; while ensemble Kalman and probabilistic transport formulations provide the practical baselines [58,59].

### 6.4. Numerical Case Study: NCD-Gated Moving-Obstacle Curriculum

We instantiate the running example as a planar double integrator with $q,p\in R^{2}$, $\dot{q}=p$, and $\dot{p}=-\nabla_{q}V\left(q;\kappa \right)-D\left(q;\kappa \right)p$. The task is to move from $q_{0}=\left(-1.15,-0.75\right)$ to $q_{g}=\left(1.15,0.75\right)$ while avoiding a circular obstacle whose center and radius drift over curriculum time. The PH energy used by the controller is

$$H\left(q,p;\kappa \right)=\frac{1}{2}{∥p∥}^{2}+\frac{\omega_{g}}{2}∥q-q_{g}{∥}^{2}{-\alpha \log(∥q-c∥}^{2}-r^{2}+\varepsilon ),$$

with anisotropic damping

$$D\left(q;\kappa \right)=\beta I+\frac{\beta_{b}}{{\left(g\left(q;\kappa \right)+\varepsilon \right)}^{2}}n\left(q\right)n{\left(q\right)}^{⊤},\;g\left(q;\kappa \right)={∥q-c∥}^{2}-r^{2},\;n\left(q\right)=\frac{q-c}{∥q-c∥}.$$

**Figure 5:**
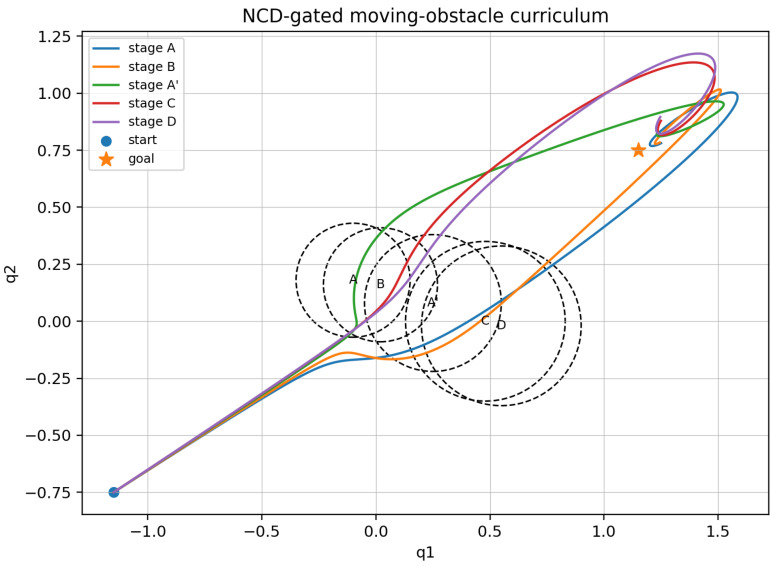
NCD-gated curriculum for the moving-obstacle double integrator. Each colored curve is a learned obstacle-avoidance stage trajectory from start to goal, and each dashed circle represents the corresponding obstacle at that stage.

The toy simulator in curriculum_learning_simulation.py uses α=0.08, $k_{\mathrm{goal}}=0.8$, β=0.8, $\beta_{b}=0.05$, ε=0.02, $dt=10^{-2}$, horizon 10, and acceleration clip 5.0. Figure 5 shows the NCD-gated curriculum from stages $\left\{A,B,A^{\prime },C,D\right\}$, along with obstacle-avoidance stage trajectory and the stage obstacles.

**Environment encoding and NCD gate**

Each environment $E_{t}$ is serialized as a canonical byte string consisting of sorted-key JSON metadata, the dynamics label, goal, sensor model, obstacle center and radius, and ordered quantized samples of the obstacle boundary. The primary compressor is LZMA/XZ preset 6. In the toy run, the admissibility threshold is $\delta_{\mathrm{safe}}=0.245$. The direct shift A→C has ${\mathrm{NCD}}_{Z}=0.265$ and is rejected; the inserted chain $A\;\to \;B\;\to \;A^{\prime }\;\to \;C\;\to \;D$ has admitted pairwise values of 0.236, 0.242, 0.237, and 0.210.

**Diagnostics**

**Figure 6:**
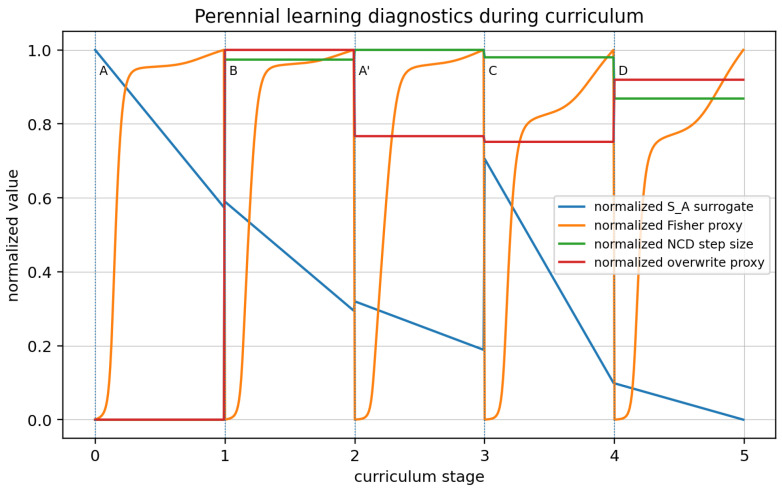
Perennial-learning diagnostics during the curriculum. The plot reports a normalized ${\hat{S}}_{A}$ surrogate, a normalized Fisher proxy, a normalized NCD step size, and a normalized overwrite proxy over the five-stage curriculum.

We monitor four quantities: a practical algorithmic-entropy surrogate ${\hat{S}}_{A}\left(t\right)$, a boundary-informative Fisher proxy, the NCD shock size between successive environments, and an overwrite proxy proportional to the conditional compressed length. Decreasing ${\hat{S}}_{A}\left(t\right)$ together with an increasing Fisher score indicates that measurements are reducing uncertainty faster than the model description grows. Figure 6 plots the perennial-learning diagnostics during the curriculum for stages $\left\{A,B,A^{\prime },C,D\right\}$ via four metrics for the toy obstacle-avoidance simulated example shown in Figure 5.

**Passivity stress test**

**Figure 7:**
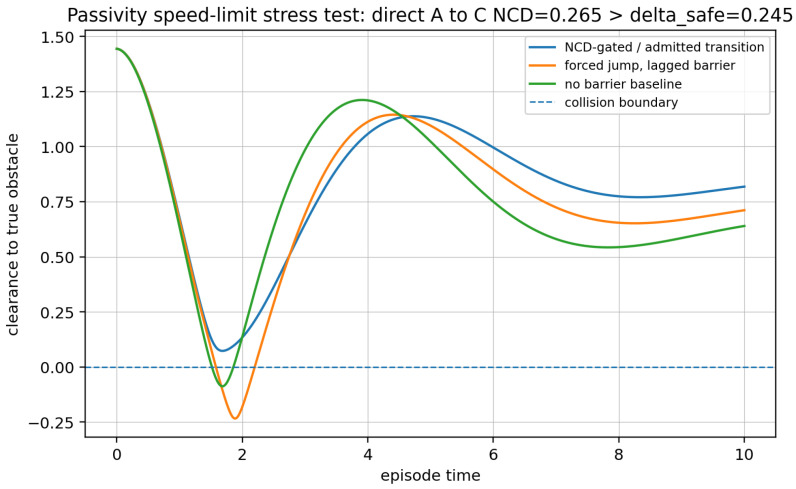
Passivity speed-limit stress test. The admitted transition maintains positive clearance, while the forced direct jump with a lagged barrier crosses the collision boundary.

To illustrate Proposition 3, we compare the NCD-gated update against a forced update in which the physical obstacle jumps from *A* to *C* while the controller barrier is still centered at *A*. Figure 7 plots the passivity speed-limit test of the toy example given above comparing four different modes. In the admitted curriculum the minimum clearance remains positive (0.043 in normalized units). In the forced jump the minimum clearance is −0.234, i.e., the true obstacle is penetrated before the lagged barrier can dissipate and redirect the trajectory. This should be read as a numerical illustration of the dissipation-bound interpretation, not as a universal benchmark.

**Compact baseline summary**

**Figure 8:**
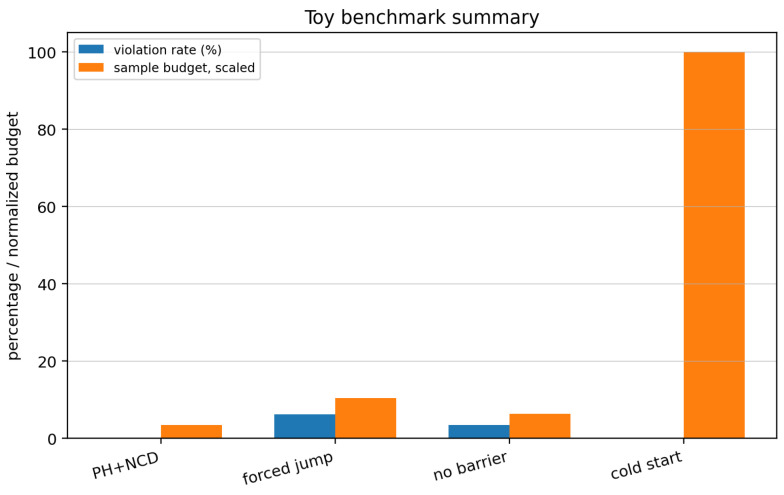
Toy benchmark summary. Sample budgets are scaled to the tallest bar; the exact numerical values are listed in Table 4.

The toy simulator compares four modes: PH + NCD, forced jump with lagged barrier, no-barrier PH goal seeking, and a cold-start PH identification baseline. PH + NCD has zero violations in this run and uses the warm-start active-block sample budget. The forced jump violates the constraint in 6.2% of time steps; the no-barrier baseline violates in 3.4%; the cold-start baseline avoids collision but uses a much larger measurement budget under the chosen cubic cold-start pipeline. Figure 8 plots a toy benchmark summary for comparing the four modes. These values are reported as a reproducible toy case study, not as a claim of broad empirical superiority over continual-learning or safe-RL baselines.

**Table 4:** Quantitative metrics of a simulated moving-obstacle benchmark. We report the validation rate, minimum clearance of the path, mean path length (MPL), and number of measurements the program required for the computations.

Method	Violation %	Min. Clearance	MPL	Measurements	Comment
PH + NCD	0.00	0.043	3.771	49	Admitted chain $A\;\to \;B\;\to \;A^{\prime }\;\to \;C\;\to \;D$
Forced jump	6.20	−0.234	3.921	148	Direct A→C exceeds $\delta_{\mathrm{safe}}$ with lagged barrier
No barrier	3.40	−0.088	4.102	90	Goal controller without log-barrier
Cold start	0.00	0.063	4.073	1419	Cubic cold-start identification baseline

## 7. Integration and Validation

### 7.1. The Perennial Inference Engine: Full Assembly

We now assemble all components into a single operational cycle.

**One inference step**

1. Observation (port $G_{\xi }u_{\xi }$): new data arrives; the demon observes molecules.
2. Transport (DPO update via J∇H): the policy pushes the current posterior toward the updated posterior via the learned transport map. This is reversible (zero Landauer cost).
3. Dissipation (Landauer cost via $R_{\xi }$): outdated beliefs are overwritten; energy $k_{B}T\ln2$ per erased bit flows through the dissipation port. This is irreversible.
4. Conservation (Casimir *C*): structural invariants—symmetries, sparsity patterns, discovered physical laws—are preserved automatically (no memory, no measurement, no Landauer cost).
5. Safety check (energy certificate): $H_{\xi }\left(x\left(t\right)\right)\leq E_{\max}\left(\tau \right)$ is verified via passivity. If approaching the boundary, $R_{\mathrm{margin}}$ increases, slowing the trajectory.

**Headline quantitative result**

Under the assumptions of Proposition 5 and Corollary 1, and relative to a cold-start baseline requiring $n\propto k^{3}\mathrm{polylog}d$ measurements, the perennial PH + DPO engine achieves $n_{\mathrm{seq}}=O\left(\Delta k_{t}\log d_{\xi }/\lambda_{⋆}\right)$ (equivalently, $\tilde{O}\left(\Delta k_{t}/\lambda_{⋆}\right)$) per step, conditional on the warm-start assumptions of Proposition 5, through the following mechanism:

$$\mathrm{Casimir}\;\mathrm{lock}\overset{\mathrm{reduces}\;\mathrm{phase}\text{-}\mathrm{space}\;\mathrm{volume}}{\to }\mathrm{lower}\;\mathrm{proposal}\;\mathrm{variance}\overset{\mathrm{DPO}\;\mathrm{transport}}{\to }\mathrm{proposal}\approx \mathrm{posterior}.$$

Table 5 provides the key differences between a static learner and a perennial PH + DPO engine. This improvement is conditional on the warm-start assumptions and the chosen cold-start baseline.

### 7.2. Numerical Validation Protocol and Broader Benchmark Agenda

**Validation Class 1: Constrained autonomy and navigation**

The toy moving-obstacle case study in Section 6.4 is the first validation class: it directly measures violation rate, minimum clearance, path length, overwrite proxy, Fisher score, and curriculum admissibility under actual simulated trajectories. A broader benchmark suite should extend this to multi-agent and multi-joint systems with progressively tightening safety/feasibility constraints [8]. The perennial agent must then handle multiple spatial scales simultaneously: local obstacle avoidance (1:1), neighborhood-level planning (1:20), district-level routing (1:400), and city-level strategy (1:80,000). Each scale has its own feasibility set F(κ), constraint complexity K(∂F), and identifiability challenges. The PH framework handles this via hierarchical energy shaping: Casimir invariants at the highest scale (road network topology, traffic laws), barrier potentials at intermediate scales (lane boundaries, intersection geometry), and dissipation shaping at the local scale (collision-avoidance margins).

**Validation Class 2: Sequential inference and data assimilation**

Changing observation operators (sensor failures, new sensors coming online) and tightening noise budgets [60,61] define the second validation class. The feasibility set then corresponds to posterior states consistent with the observation model; sensor failure expands the feasible posterior in an unexplored direction. In that regime, the relevant empirical baselines include replay-style continual-learning policies, EWC/GEM-style parameter protection, ensemble Kalman methods, and safe control and barrier-function baselines. We do not claim superiority over those families here; rather, the present paper supplies the PH bookkeeping and the toy case study that such a large benchmark should evaluate.

**Safety-specific evaluation metrics**

(i) Constraint-violation frequency.
(ii) Minimum distance to constraint boundary over trajectory.
(iii) Energy-budget utilization $H\left(t\right)/E_{\max}$.
(iv) Casimir-invariant drift |C(x(t))−C(x(0))|.
(v) Hausdorff distance between $F_{\mathrm{eff}}$ and F(κ).

## 8. Discussion, Limitations, and Conclusions

We have developed a unified framework for perennial machine learning grounded in three classical pillars: Kolmogorov complexity (learning as compression), Landauer accounting (irreversible overwrite has a physical lower bound in concrete devices and a formal bookkeeping role in the abstract model), and port-Hamiltonian dynamics (the learning engine has structure). The Maxwell/Szilard analogy motivates the separation between reversible transport and irreversible overwrite, but the formal content of this paper consists of the explicit set of propositions and assumptions. The information-distance framework provides a computable geometry for measuring learning progress and designing safe curricula. Differential Policy Optimization supplies one transport law, imported from [42], with a regret bound interpretable as excess overwrite cost. The resulting perennial inference engine separates zero-cost reversible inference from costly irreversible forgetting by construction, enforces safety through energy certificates and Casimir invariants, and achieves amortized measurement complexity $n_{\mathrm{seq}}=\tilde{O}\left(\Delta k\log d_{\xi }/\lambda_{⋆}\right)$ relative to a chosen cold-start baseline, conditional on warm-start initialization (Proposition 5 and Corollary 1).

### 8.1. Thermodynamic Learning by Lifting, Reduction, and PH Model Enrichment

A complementary thermodynamic interpretation of learning is complexity reduction through the recognition of a pattern in the PH phase portrait. In this view, the data are not only observations to be compressed but also a family of trajectories generated by a PH vector field. Learning occurs when this family is recognized as the projection of a lower-dimensional or more structured PH dynamics. This is common in nonequilibrium and rate thermodynamics: one often lifts the original variables to tangent, cotangent, or contact-type spaces, studies the lifted dynamics, and then identifies a reduced dynamics whose phase portrait captures the emergent pattern. The classical Lagrange multiplier construction can be read in this way: the constrained problem is lifted to an augmented space in which constraints become geometric variables, after which the reduced extremal dynamics reveals the effective law.

This perspective is consistent with the perennial-learning framework. The PH learner can either compress a phase portrait by reducing variables when a coherent pattern is discovered or enrich the model when the current coarse variables no longer close the dynamics. The latter is essential in thermodynamic modeling: a simple-fluid model may ignore microscopic configuration variables, whereas a complex-fluid model must promote molecular orientation, conformation, or internal stress into the state. In our notation, this enrichment may increase K(ξ) or $L_{Z}\left(\xi \right)$ initially but can reduce the residual uncertainty enough to lower the total description length. Thus, reduction and enrichment are two sides of the same compression principle: reduce when the lifted phase portrait reveals a lower-order invariant and extend when omitted microscopic variables become macroscopically active. From the viewpoint of algorithmic statistics, this is also a model-selection question about which structured description best balances fit and complexity, in the spirit of Kolmogorov structure functions [4]. A full theory of automated PH lifting/reduction remains open.

### 8.2. Limitations

1. Proxy calibration is local and task-family dependent. Exact *K* is never optimized directly. Every implementable program statement uses $L_{Z}$ or another explicit code proxy, and Assumption 3 is deliberately restricted to the finite serialized model family actually visited by the curriculum.
2. The Fisher codelength proxy is local. Proposition 4 is a Laplace/MDL-style curvature diagnostic; it can fail for multimodal, aliased, or strongly misspecified posteriors.
3. The $k^{3}\;\to \;\Delta k$ comparison is conditional. Corollary 1 is relative to a chosen cold-start baseline and depends on warm start, active-block drift, and informative local curvature. It is not a universal law of perennial learning.
4. NCD admissibility is a screen, not a guarantee. Curriculum gating is compressor- and representation-dependent. It becomes a validated metric only after correlation with transfer cost, overwrite cost, or safety violations is measured in the task family.
5. Numerical stiffness and solver dependence remain practical constraints. Barriers near the boundary require regularization and careful time stepping, and DPO is one plausible transport solver rather than a theorem of necessity.

More broadly, the present framework also incorporates the algorithmic information-theoretic view of inductive learning and cognition: if learning is compression under structural constraints, then perennial adaptation can be considered a controlled form of inductive inference with memory and overwrite budgets [41,50].

### 8.3. Open Problems

1. Adaptive Casimir selection. Can the system learn which invariants to protect? If a constraint has not changed in *N* curriculum steps, $K(\kappa_{t+N}|\kappa_{t})\approx 0$, does this suggest promotion to Casimir status?
2. Computable *K* proxies. Can we replace Kolmogorov complexity with practical compression-based surrogates (NCD, minimum description length) for the Landauer cost, $\Omega_{\mathrm{life}}$, and NID-based curriculum [3,4,5]?
3. The safety–identifiability frontier. Does the optimal boundary dissipation β minimize the total Landauer cost, balancing extra-measurement cost (poor identifiability at high β) against constraint-violation recovery cost (at low β)?
4. Algorithmic emergence detection. Can the agent detect phase transitions in its own learning dynamics—moments where $S_{\;A}$ drops discontinuously, signaling qualitative discovery [44,62]?
5. Tighter regret under PH structure. Does the $O(K^{5/6})$ bound tighten when DPO is applied to a system with known PH structure?
6. Quantum extension. How can we extend the framework to quantum inference, where *K* of pure quantum states replaces classical *K* ([32], §8.7)?

## Figures and Tables

**Table 1:** A nomenclature mapping between the Maxwell’s Demon and the Learning Agent.

Demon Component	Learning-Agent Analog
Demon’s memory	Meta-parameters η (policy/design variables)
Measuring molecule speed	Observing data
Sorting fast/slow	Updating posterior
Memory erasure	Overwriting old beliefs ($\Omega_{\mathrm{life}}$)
Heat-bath temperature *T*	Environment stochasticity
Szilard cycle	One episode of online learning

**Table 2:** Thermodynamic interpretation of PH terms.

PH Term	Thermodynamic Role	Kolmogorov Cost
J∇H (symplectic)	Reversible inference; reorganizes beliefs	Zero Landauer cost
$-R\nabla_{p}H$ (dissipation)	Irreversible forgetting; erases outdated beliefs	$k_{B}T\ln2$ per erased bit
$G_{\xi }u_{\xi }$ (control port)	Observation intake; new data enters the system	Information gain ≤K(u)
$\Sigma \;\;dW_{t}$ (noise port)	Environmental stochasticity	Irreducible uncertainty

**Table 3:** Safety-mechanism tradeoffs.

Type	Enforcement	Landauer Cost	Use for
Casimir ({C,H}=0)	Permanent, exact	Zero	Conservation laws, hard actuator limits
Barrier ($V_{\mathrm{barrier}}$)	Soft, adjustable	K(ΔV) per update	Moving obstacles, tightening clearances
Dissipation ($R_{\mathrm{margin}}$)	Graduated slowdown	Moderate (operating cost)	Safety margins, approach speed limits

**Table 5:** Static learner vs. perennial PH + DPO engine.

Component	Static Learner	Perennial PH + DPO
Proposal *Q*	Fixed product distribution	Learned transport $\mu_{K}$ via DPO
Search space	Discrete subset $\Omega_{k}$	Casimir-locked phase space
Safety	Post-hoc constraint check	Energy certificate + barrier + dissipation
Scaling	$n=\Theta (k^{3}\mathrm{polylog}d)$ (global)	$n=\tilde{O}\left(\Delta k/\lambda_{⋆}\right)$ (warm-start, conditional)
Forgetting cost	Uncontrolled (catastrophic)	Budgeted by $\Omega_{\mathrm{life}}\propto \Delta K$
Structure	None	Casimir invariants (zero Landauer cost)
Curriculum	None	NID/NCD-guided feasibility-set evolution

## Data Availability

No new data were created or analyzed in this study. Data sharing is not applicable to this article.

## Appendix A. Reproducibility Details for the NCD-Gated Toy Case Study

**Primary compressor**

This manuscript uses LZMA/XZ preset 6 as the primary compressor for $L_{Z}$ and ${\mathrm{NCD}}_{Z}$ in the toy study. Sensitivity checks use gzip/zlib level 6 and parameter-vector encoding.

**Canonical serialization**

Each environment encoding begins with sorted-key JSON metadata containing the dynamics label, mass, goal, sensor model, barrier type, damping type, grid size, obstacle center, obstacle radius, and stage label. This header is followed by repeated ordered text records for the obstacle geometry and ordered boundary samples. Strings are UTF-8 encoded, and floating-point values are quantized before serialization.

**Toy simulation hyperparameters**

The numerical study in Section 6.4 uses horizon 10.0, step size dt=0.01, barrier weight α=0.08, goal-spring coefficient $k_{\mathrm{goal}}=0.8$, isotropic damping β=0.8, margin damping $\beta_{b}=0.05$, smoothing ε=0.02, and acceleration clip 5.0. The curriculum scenes are

$$A=\left(-0.10,0.18,0.25\right),\;B=\left(0.02,0.16,0.25\right),\;A^{\prime }=\left(0.25,0.08,0.30\right),\;C=\left(0.48,0.00,0.35\right),\;D=\left(0.55,-0.02,0.35\right),$$

where each tuple has an obstacle center $\left(c_{1},c_{2}\right)$ and radius *r*.

**Curriculum thresholds and outcomes**

The toy run uses $\delta_{\mathrm{safe}}=0.245$. The admitted pairwise NCD values are 0.236, 0.242, 0.237, and 0.210, while the direct A→C jump has ${\mathrm{NCD}}_{Z}=0.265$ and is rejected by the screen. The accompanying JSON file toy_benchmark_results.json records the exact table values used in Section 6.4.
